# Citicoline Protects Auditory Hair Cells Against Neomycin-Induced Damage

**DOI:** 10.3389/fcell.2020.00712

**Published:** 2020-08-31

**Authors:** Zhenhua Zhong, Xiaolong Fu, He Li, Jie Chen, Maohua Wang, Song Gao, Liyan Zhang, Cheng Cheng, Yuan Zhang, Peipei Li, Shasha Zhang, Xiaoyun Qian, Yilai Shu, Renjie Chai, Xia Gao

**Affiliations:** ^1^Jiangsu Provincial Key Medical Discipline (Laboratory), Department of Otolaryngology Head and Neck Surgery, Nanjing Drum Tower Hospital Clinical College of Nanjing Medical University, Nanjing, China; ^2^Department of Otolaryngology, Head and Neck Surgery, The Affiliated Hospital of Yangzhou University, Yangzhou University, Yangzhou, China; ^3^MOE Key Laboratory for Developmental Genes and Human Disease, Jiangsu Province High-Tech Key Laboratory for Bio-Medical Research, Institute of Life Sciences, Southeast University, Nanjing, China; ^4^Department of Otolaryngology-Head and Neck Surgery, First Affiliated Hospital of Wenzhou Medical University, Wenzhou, China; ^5^Department of Otolaryngology, Head and Neck Surgery, Xiangya School of Medicine, Central South University, Changsha, China; ^6^Department of Otolaryngology, Affiliated People’s Hospital of Jiangsu University, Zhenjiang, China; ^7^School of Life Sciences, Shandong University, Jinan, China; ^8^ENT Institute and Department of Otorhinolaryngology, Eye & ENT Hospital, State Key Laboratory of Medical Neurobiology, Institute of Biomedical Sciences, NHC Key Laboratory of Hearing Medicine, Fudan University, Shanghai, China; ^9^Co-innovation Center of Neuroregeneration, Nantong University, Nantong, China; ^10^Institute for Stem Cell and Regeneration, Chinese Academy of Sciences, Beijing, China; ^11^Beijing Key Laboratory of Neural Regeneration and Repair, Capital Medical University, Beijing, China

**Keywords:** citicoline, hair cell, apoptosis, reactive oxygen species, aminoglycosides

## Abstract

Aminoglycoside-induced hair cell (HC) loss is one of the most important causes of hearing loss. After entering the inner ear, aminoglycosides induce the production of high levels of reactive oxygen species (ROS) that subsequently activate apoptosis in HCs. Citicoline, a nucleoside derivative, plays a therapeutic role in central nervous system injury and in neurodegenerative disease models, including addictive disorders, stroke, head trauma, and cognitive impairment in the elderly, and has been widely used in the clinic as an FDA approved drug. However, its effect on auditory HCs remains unknown. Here, we used HC-like HEI-OC-1 cells and whole organ explant cultured mouse cochleae to explore the effect of citicoline on aminoglycoside-induced HC damage. Consistent with previous reports, both ROS levels and apoptosis were significantly increased in neomycin-induced cochlear HCs and HEI-OC-1 cells compared to undamaged controls. Interestingly, we found that co-treatment with citicoline significantly protected against neomycin-induced HC loss in both HEI-OC-1 cells and whole organ explant cultured cochleae. Furthermore, we demonstrated that citicoline could significantly reduce neomycin-induced mitochondrial dysfunction and inhibit neomycin-induced ROS accumulation and subsequent apoptosis. Thus, we conclude that citicoline can protect against neomycin-induced HC loss by inhibiting ROS aggregation and thus preventing apoptosis in HCs, and this suggests that citicoline might serve as a potential therapeutic drug in the clinic to protect HCs.

## Introduction

Sensorineural hearing loss remains a serious sensory disorder worldwide. Once the hair cells (HCs) of the inner ear are damaged, sensorineural hearing loss is permanent due to the inability of mammalian HCs to regenerate. The causes of hearing loss include many factors, such as noise, age, and ototoxic drugs, all of which can induce apoptosis in HCs (Prasad and Bondy, 2020). Damage caused by aminoglycosides, which are the most commonly used ototoxic drugs, is a major cause of HC death (Waguespack and Ricci, 2005). Therefore, it is important to investigate the molecular mechanism behind aminoglycoside-induced auditory sensory cell damage and to seek effective drugs for preventing and treating aminoglycoside-induced deafness. Several studies have suggested that aminoglycosides induce intrinsic apoptosis of HCs through oxidative stress (Mangiardi et al., 2004; Coffin et al., 2013; Sun et al., 2014, 2015; Liu et al., 2016), while others have reported that the accumulation of reactive oxygen species (ROS) plays an important role in the death of HCs (Clerici et al., 1996; Choung et al., 2009). ROS can be cleared by physiological cellular processes; however, they are harmful when their concentration exceeds the cell’s capacity to remove them (He et al., 2020). Thus, the survival of HCs requires a balance between oxidative stress and anti-oxidation (Franco and Cidlowski, 2009; Filomeni et al., 2015), suggesting that preventing the accumulation of ROS might be a potential treatment for preventing ototoxicity.

Citicoline, a nucleoside derivative, is an essential endogenous intermediate in phosphatidylcholine biosynthetic pathways. Citicoline is widely distributed in the body, and it can easily cross the blood-brain barrier and penetrate brain cells to provide neural protection (Secades and Lorenzo, 2006). It has been widely demonstrated that citicoline can activate the biosynthesis of phospholipids in neuronal membranes, and this stimulates brain metabolism and the neurotransmitter system (Secades and Lorenzo, 2006). It also plays an important role in promoting the recovery of brain function and in awakening from sleep (Adibhatla et al., 2002). When used as a long-term medication, citicoline is a safe and well-tolerated drug without significant systemic side effects. Citicoline can be used to treat addictive disorders (Wignall and Brown, 2014), stroke, head trauma (Saver, 2008), and cognitive impairment in the elderly (Cotroneo et al., 2013), and it is also effective in the treatment of glaucoma (Parisi et al., 2008). In addition, citicoline has anti-apoptotic effects by disrupting mitochondria-dependent cell death mechanisms and promoting the regeneration of nerve axons, and it can protect retinal ganglion cells from damage (Oshitari et al., 2002).

There have been no reports on whether citicoline can protect auditory HCs from aminoglycoside-induced injury up to now. In our study, we used the HC-like House Ear Institute Organ of Corti 1 (HEI-OC-1) cell line along with explant cultured cochlear HCs to establish an *in vitro* neomycin-induced damage model in auditory HCs with the aim to investigate the potential protective effect of citicoline in auditory HCs.

## Materials and Methods

### Animals

All animal procedures were performed according to protocols approved by the Animal Care and Use Committee of Southeast University, and all efforts were made to minimize the number of animals used and to prevent their suffering.

### Cell Cultures and Tissue Cultures

Consistent with previous studies, we used HEI-OC1 (House Ear Institute-organ of Corti 1) cells derived from long-term cultures of Immortomouse cochleae. HEI-OC1 cells express *Atoh1, Prestin, Myo7a*, and other cellular markers specific for auditory sensory HCs when cultured either under permissive or non-permissive conditions. The cells were cultured in DMEM containing 10% fetal bovine serum and 100 IU/ml penicillin (A0166, Sigma-Aldrich, St. Louis, United States) at 37°C and 5% CO_2_ (Kalinec et al., 2003; He et al., 2020) and sub-cultured at 80% confluency using 0.25% trypsin/EDTA (25200056, Life Technologies, Waltham, MA, United States). Neomycin (N6386, Sigma-Aldrich) was used at a final concentration of 10 mM to damage the HEI-OC-1 cells. Citicoline (C0256, Sigma-Aldrich) was used at a final concentration of 10 μM to treat the HEI-OC-1 cells.

Cochleae were dissected from postnatal day (P)3 mice and cultured as previously reported (Chen et al., 2013), The explant cultured tissue was pretreated with 10 μM citicoline for 12 h, then 0.5 mM neomycin was added for 12 h to damage the HCs. After removal of the neomycin, the tissues were recovered in serum-free medium for an additional 12 h together with 10 μM citicoline. Animal experiments were conducted in accordance with the guidelines of the Committee of Animal Protection and Utilization of Southeast University and were approved by the Animal Experimental Ethics Committee of Southeast University.

### Real-Time PCR

Total RNA was extracted from HEI-OC-1 cells or whole cochleae with Trizol reagent (PR910, Protein Biotechnology, Beijing, China), and the integrity of the RNA samples was evaluated by OD260/280 measurements. cDNA was obtained using the Revertaid First Strand cDNA Synthesis Kit (K1622, Thermo Fisher Scientific) according to the manufacturer’s protocol. Real-time PCR was performed on a Biosystems CFX96 real-time PCR system (Bio-Rad, Hercules, CA, United States) using SYBR Green qRT-PCR Master Mix (4913850001, Roche Life Science, Basel, Switzerland). The primer sequences are listed in Table 1. The qRT-PCR conditions were as follows: initial denaturing for 15 s at 95°C followed by 40 cycles of denaturation at 95°C for 15 s, annealing at 60°C for 60 s, and extension at 72°C for 20 s. The mRNA expression values of the genes of interest were normalized to the mRNA expression of *Gapdh*. The results were calculated using the comparative cycle threshold (ΔΔCt) method.

**TABLE 1:** PCR sequences used in the experiments.

Gene	Forward sequence	Reverse sequence
Caspase-3	AATCATGCCATTTGCCCAGC	CTCAAGTGTGTAGGGGGAGG
Caspase-8	AGCCTATGCCACCTAGTGAT	GGAGAGCTGTAACCTGTCGC
Bcl-2	GGTGAACTGGGGGAGGATTG	AGAGCGATGTTGTCCACCAG
Caspase-9	CCTAGTGAGCGAGCTGCAAG	ACCGCTTTGCAAGAGTGAAG
Bax	TGAAGACAGGGGCCTTTTTG	AATTCGCCGGAGACACTCG
Alox15	TCGGGACTCGGAAGCAGAAT	CCCATCGGTAACAGGGGAAC
Gsr	TGCACTTCCCGGTAGGAAAC	GATCGCAACTGGGGTGAGAA
Sod1	GGAGCAAGGTCGCTTACAGA	AGTGACAGCGTCCAAGCAAT
Glrx	AGTCTGGAAAGGTGGTCGTG	CCATTAGCATGGCTGGACGA
GADPH	GCAAGAGAGAGGCCCTCAG	TGTGAGGGAGATGCTCAGTG

### Cell Number Analysis

HEI-OC-1 cells were incubated in 96-well plates for 24 h at a concentration of 2,000 cells/well in three replicates, and different drugs were added (controls received a similar volume of DMEM). The Cell Counting Kit (CCK-8; CC201, Protein Biotechnology, Beijing, China) was used to determine the cell viability at different time points after incubation or at different concentrations. After exposing the cochleae to citicoline and/or neomycin, the immunostained cells were quantified per 100 μm in all three turns of the cochlea. The numbers of positive cells were counted in equal lengths from the apical to the basal turns of the cochlea.

### Western Blot

The HEI-OC-1 cell line and cochlear tissue were lysed with ice-cold RIPA lysis buffer (PP109, Protein Biotechnology) plus Phosphatase Inhibitor Cocktails (04693132001, Roche) for 30 min at 4°C. Protein concentrations were determined using a BCA Protein Quantification Kit (PP202, Protein Biotechnology) according to the manufacturer’s instructions. Equal amounts of protein were loaded onto a 12% Tris-glycine SDS-PAGE gel, separated at 120 volts for 1.5 to 2 h, and then transferred to a nitrocellulose membrane and blocked with 5% milk in PBST [1 × PBS with 0.1% Triton X-100 (Solarbio, 1109F0521)] buffer. Cleaved caspase 3 was evaluated using anti-cleaved caspase 3 rabbit monoclonal antibody (1:1,000 dilution, 9664S, Cell Signaling Technology), and β-actin was measured using a mouse monoclonal antibody (1:5,000 dilution, ab119716, Abcam, Cambridge, United Kingdom). Peroxidase-conjugated goat anti-rabbit or anti-mouse immunoglobulin G (ab6789 and ab6721, Abcam) was used as the secondary antibody. The proteins were detected using a SuperSignal West Dura chemiluminescent substrate kit (34075, Thermo Scientific) according to the manufacturer’s instructions. Semi-quantification of the western blot results was done by measuring the intensities of the bands using ImageJ.

### Immunofluorescence

Anti-cleaved caspase 3 antibody (1:400 dilution, 9664S, Cell Signaling Technology), MitoSOX Red (M36008, Life Technologies), tetramethylrhodamine ethyl ester perchlorate (TMRE, Sigma-Aldrich), anti-Myo7A antibody (1:1,000 dilution, 25–6790, Proteus Bioscience), anti-voltage dependent anion channel1 (VDAC1) polyclonal antibody (1:200 dilution, 10866-1-AP, Proteintec), and DAPI (1:1,000 dilution, C0060, Solarbio) were used to analyze apoptotic cells, measure ROS, stain HCs, measure mitochondrial number, and stain nuclei, respectively. Briefly, cells and cochlear tissues were fixed in 4% paraformaldehyde (158127, Sigma-Aldrich) for 1 h then washed three times with PBST [1 × PBS with 0.1% Triton X-100 (Solarbio, 1109F0521)] and incubated for 1 h in blocking medium (PBS with 10% heat-inactivated donkey serum, 1% Triton X-100, 1% BSA, and 0.02% sodium azide at pH 7.2) at room temperature. The samples were marked with primary antibody diluted in PBT-1 (PBS with 10% Triton X-100, 5% heat-inactivated donkey serum, 1% BSA, and 0.02% sodium azide at pH 7.2) for overnight at 4°C. After washing three times with PBST, the samples were marked with the secondary antibody diluted in PBT-2 (PBS with 1% BSA and 0.1% Triton X-100 at pH 7.2) for 1 h. The samples were washed again three times and were imaged by confocal fluorescence microscopy (Leica SP5, Heidelberg, Germany).

The TUNEL Kit (11684817910, Roche, Indianapolis, IN, United States) was used to detect apoptotic cells following the manufacturer’s instructions. TMRE was used for measuring the mitochondrial membrane potential (MMP), and Mito-SOX Red was used to analyze ROS levels. Briefly, the culture medium was removed from the dish and the samples were washed with PBS. The samples were then cultured in DMEM containing Mito-SOX Red or TMRE at 37°C for 30 min. The samples were then washed in pre-warmed PBS and imaged with a confocal microscope (LSM700; Zeiss, Heidenheim, Germany).

### Flow Cytometry

Annexin V-FITC and propidium iodide (C1062, Beyotime) were used for apoptosis analysis following the manufacturer’s instructions. After treating the HEI-OC-1 cells with citicoline and/or drugs, the cells were trypsinized and collected and then washed twice with PBS and resuspended in binding buffer at a concentration of 1 × 10^6^ cells/ml. Annexin V-FITC and propidium iodide were added and gently mixed with the cells and incubated at room temperature for 10–20 min in the dark. Cells were analyzed as quickly as possible by flow cytometry (FACSCanto, BD, San Jose, CA, United States).

Mito-SOX Red and TMRE were used to analyze ROS production and to measure the MMP, respectively. After treating the HEI-OC-1 cells with citicoline and/or neomycin, the cells were trypsinized, collected, and resuspended in pre-warmed solution containing Mito-SOX Red or TMRE for 10 min. Following this, the cells were washed with PBS and analyzed by flow cytometry. All experiments were repeated at least three times.

### Statistical Analysis

All data are shown as mean ± standard deviation (SD), and all experiments were repeated at least three times. Statistical analyses were performed using Microsoft Excel and GraphPad Prism 6 software (La Jolla, CA, United States). For the cochlear tissue culture experiments, “n” represents the number of independent cochleae, and for all HEI-OC-1 cell culture experiments “n” represents the number of replicates. When comparing two groups, a two-tailed, unpaired Student’s *t*-test was used to determine statistical significance. When comparing more than two groups, one-way ANOVA was used followed by Dunnett’s multiple comparison test. A *p*-value < 0.05 was considered to be statistically significant.

## Results

### The Survival of HEI-OC-1 Cells Is Affected by Neomycin and Citicoline Treatment

To select the proper conditions to induce cell death in HEI-OC-1 cells, we exposed HEI-OC-1 cells to different doses of neomycin (1–20 mM) for different times (0–24 h). We found that the viability of HEI-OC-1 cells decreased gradually with increasing neomycin doses and time, and 50–60% of the HEI-OC-1 cells were alive after being treated with 10 mM neomycin for 24 h. Thus we chose this condition to induce HEI-OC-1 cell damage (Supplementary Figures S1A,B). Because there are no reports of using citicoline to protect against ototoxic drug-induced HC loss, we first determined the appropriate dose and treatment time of citicoline in HEI-OC-1 cells before neomycin exposure. We pre-treated the HEI-OC-1 cells with different concentrations of citicoline (1, 10, 100 μM, 1 mM, and 2 mM) for different times (6, 12, and 24 h, respectively) and then treated the cells with 10 mM neomycin together with citicoline (the same concentration as pre-treatment) for 24 h. The CCK8 results showed that there was no protective effect of citicoline when added 6 h in advance, but there was a clear protective effect when citicoline was added 12 or 24 h in advance. Additionally, the viability of HEI-OC-1 cells gradually increased with low concentrations of citicoline, but once the concentration of citicoline was higher than 10 μM the viability of HEI-OC-1 cells began to decrease. We also found that there was no significant difference in cell viability with the different pretreatment times (12 and 24 h). Therefore, we chose 10 μM citicoline pretreatment for 12 h as the optimal treatment condition in this study (Supplementary Figures S1C–F).

### Citicoline Treatment Protects HCs in Whole Organ Cultured Cochleae Against Neomycin Injury

**FIGURE 1:**
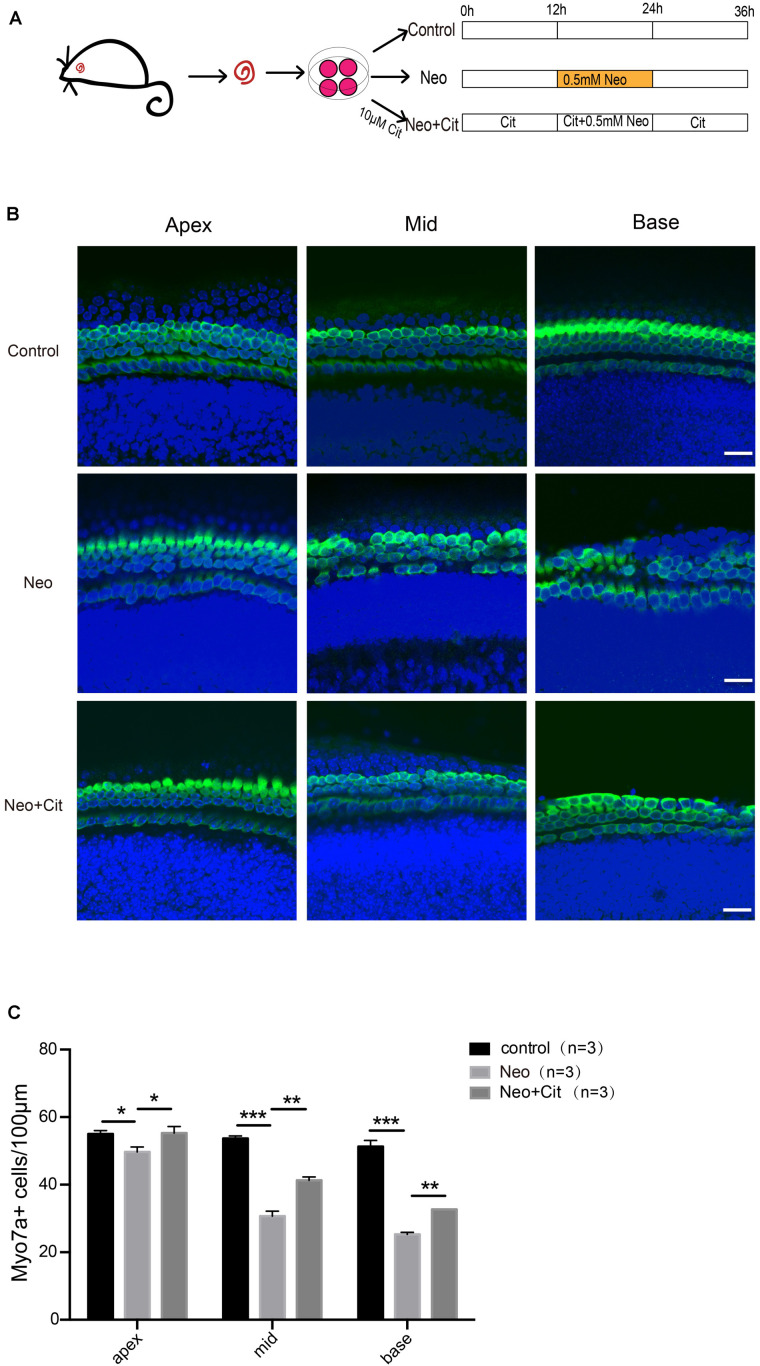
Citicoline protects against apoptosis in cochlear HCs after neomycin injury. **(A)** Schematic diagram of drug addition in tissue culture. **(B)** Immunofluorescence staining with Myo7A (green) and DAPI (blue) in the apical, middle, and basal turns of the cochlear explant cultures with different treatments. **(C)** Quantification of the numbers of Myo7A and DAPI double-positive cells in **(B)**. Data are shown as mean ± SD. *p < 0.05, **p < 0.01, ***p < 0.001. Scale bars = 20 μm.

Cochleae from P3 mice were used to investigate the role of citicoline in cochlear HCs after neomycin treatment (Figure 1A). We first used Myo7A and DAPI staining to observe the changes in the numbers of HCs in the apical, middle, and basal turns of the cochlea after different treatments. We found that the number of Myo7A and DAPI double-positive cells per 100 μm of cochlear length of the apical, middle, and basal turns was significantly lower in the neomycin-only group than in the undamaged controls (Figures 1B,C). In contrast, the number of double-positive cells was significantly increased in the citicoline-treated group compared with the neomycin-only group (Figures 1B,C).

### Citicoline Reduces Apoptosis in Cochlear HCs After Neomycin Exposure

**FIGURE 2:**
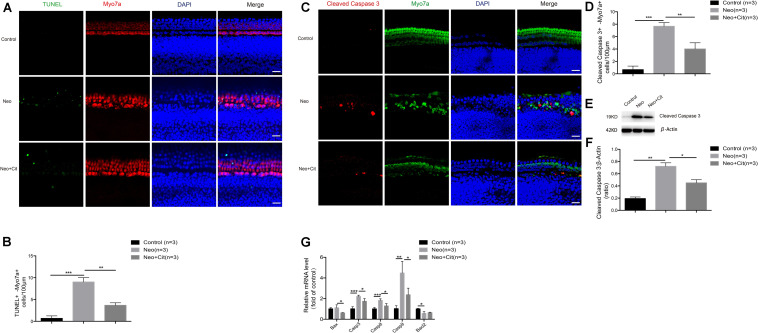
Citicoline reduces the expression of apoptotic factors in cochlear HCs after neomycin exposure. **(A)** Immunofluorescence staining with TUNEL and Myo7A in the middle turn of the cochlea after different treatments. **(B)** Quantification of the numbers of TUNEL and Myo7A double-positive cells in **(A)**. **(C)** Immunofluorescence staining of cleaved caspase 3 and Myo7A in the middle turn of the cochlea after different treatments. **(D)** Quantification of the numbers of cleaved caspase 3 and Myo7A double-positive cells in **(C)**. **(E)** Western blot showing that the amount of cleaved caspase 3 in the neomycin-only group was higher than in the undamaged control. The amount of cleaved caspase 3 induced by neomycin was significantly reduced by pretreatment with citicoline. **(F)** Quantification of the western blot in **(E)**. **(G)** The mRNA levels of five apoptosis-related genes were analyzed by qRT-PCR after neomycin and citicoline treatment normalized to *GAPDH* and presented as the fold change compared to control levels. Data are shown as mean ± SD. *p < 0.05, **p < 0.01, ***p < 0.001. Scale bars = 20 μm.

Next, we explored the role of citicoline in neomycin-induced HC injury. Previous studies have shown that cleaved caspase 3 and TUNEL can be used as markers for apoptosis induced by aminoglycosides (Matsui et al., 2002; Coffin et al., 2013; He et al., 2014). Therefore, immunofluorescence staining was used to evaluate the expression of cleaved caspase 3 and TUNEL in cochlear HCs after citicoline pretreatment. The results showed that the numbers of cleaved caspase 3-positive cells and TUNEL-positive cells per 100 mm of the cochlea in the middle turn were significantly increased in the neomycin-treated group compared with the undamaged controls (Figures 2A–D). Moreover, the citicoline-pretreated cochleae showed significantly lower numbers of caspase 3-positive cells and TUNEL-positive cells than the neomycin-only group (Figures 2A–D). Western blot results also showed that the expression levels of cleaved caspase 3 in the neomycin-only group were higher than in the undamaged controls (Figures 2E,F), while they were significantly decreased in the citicoline-treated group compared with the neomycin-only group (Figures 2E,F).

We also performed quantitative real-time polymerase chain reaction (qRT-PCR) to investigate the expression of apoptosis-related genes in the cochlea after citicoline treatment. Compared with the undamaged controls, the expression of the intrinsic and extrinsic pro-apoptotic genes *Casp3*, *Casp8*, and *Casp9* was significantly increased in the neomycin-only group, while the expression of the anti-apoptotic gene *Bcl2* was significantly decreased and expression of the pro-apoptotic gene *Bax* was not significantly different (Figure 2G). Notably, citicoline treatment significantly downregulated the expression of the pro-apoptotic genes *Bax*, *Casp3*, *Casp8*, and *Casp9* after neomycin exposure (Figure 2G). Together, these results suggest that citicoline reduces apoptosis in cochlear HCs after neomycin exposure.

### Citicoline Reduces Apoptosis in HEI-OC-1 Cells After Neomycin Exposure

**FIGURE 3:**
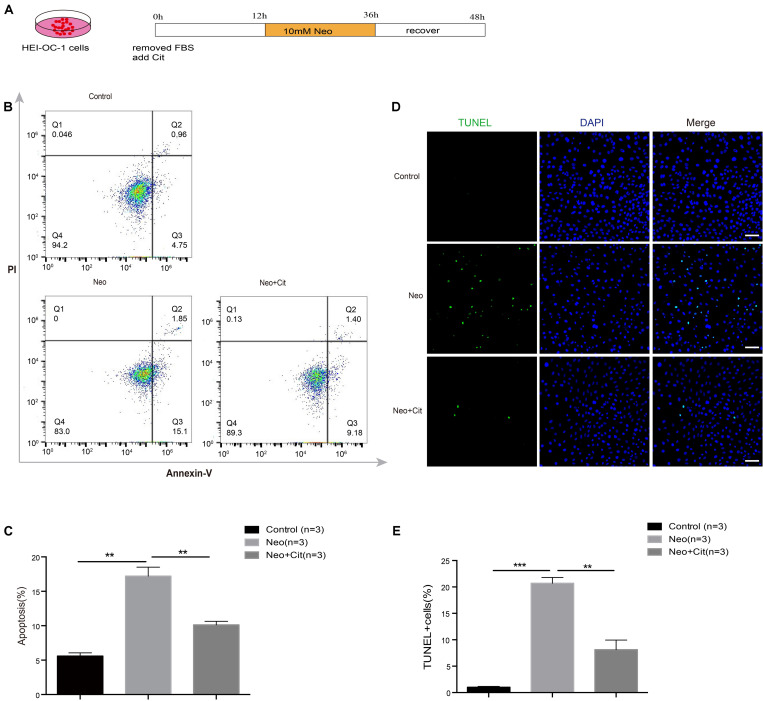
Citicoline protects against apoptosis in HEI-OC-1 cells after neomycin exposure. **(A)** Schematic diagram of citicoline (Cit) and neomycin addition in cell culture. **(B)** Apoptosis analysis by flow cytometry after different treatments. The upper right quadrants, lower right quadrants, and lower left quadrants of the images represent late apoptotic cells, early apoptotic cells, and live cells, respectively. **(C)** Flow cytometry results showing that the percentages of apoptotic cells after neomycin treatment were significantly higher compared with the undamaged controls. The amount of apoptosis induced by neomycin was significantly reduced by pretreatment with citicoline. **(D)** TUNEL and DAPI double staining showing the apoptotic HEI-OC-1 cells after different treatments. **(E)** The number of TUNEL/DAPI double-positive cells after neomycin exposure was significantly reduced by treatment with citicoline. Data are shown as mean ± SD. *p < 0.05, **p < 0.01, ***p < 0.001. Scale bars = 20 μm.

To investigate the role of citicoline in neomycin-induced death in HEI-OC-1 cells, the cells were pretreated with 10 μM citicoline for 12 h and then treated with 10 mM neomycin along with citicoline for 24 h and allowed to recover in culture medium for another 12 h together with citicoline (Figure 3A). We labeled the dead cells with propidium iodide and labeled the cells undergoing apoptosis with Annexin V. The percentage of apoptotic cells was significantly higher after neomycin treatment compared to the undamaged group, while the percentage was significantly reduced in the citicoline-treated group compared with the neomycin-only group (Figures 3B,C). To verify this result, we performed TUNEL staining to further detect apoptosis in HEI-OC-1 cells. TUNEL staining showed that the proportion of TUNEL-positive cells in the neomycin-induced group was significantly higher than in the undamaged group, while the citicoline-treated group showed significantly lower percentages of TUNEL-positive cells compared with the neomycin-only group (Figures 3D,E). Together, these results show that citicoline decreases apoptosis in HEI-OC-1 cells after neomycin exposure.

### Citicoline Reduces the Expression of Apoptotic Factors in HEI-OC-1 Cells After Neomycin Injury

**FIGURE 4:**
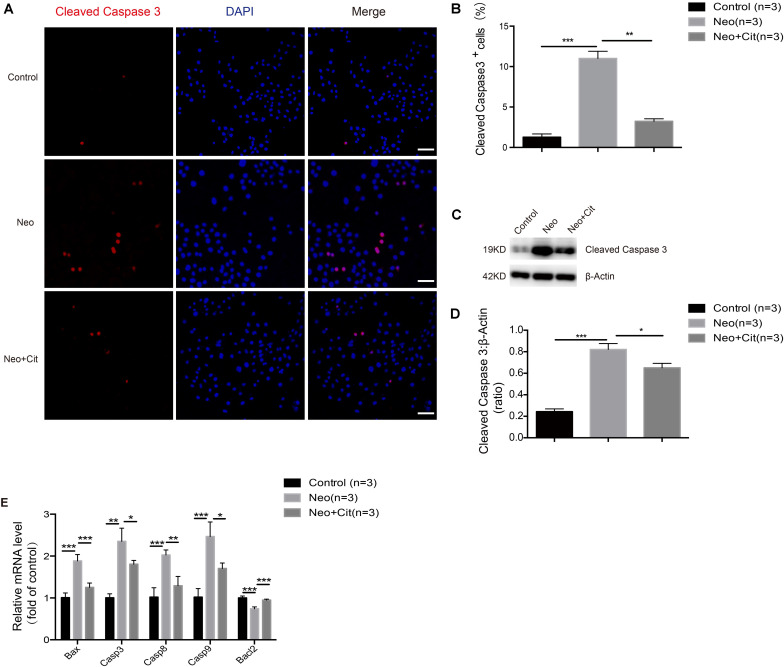
Citicoline reduces the expression of apoptotic factors in HEI-OC-1 cells after neomycin exposure. **(A)** Cleaved caspase 3 and DAPI double staining confirmed the apoptotic cells after different treatments. **(B)** Quantification of the numbers of cleaved caspase 3 and DAPI double-positive cells in **(A)**. **(C)** Western blot showing that the amount of cleaved caspase 3 in the neomycin-only groups was higher than in the undamaged controls. The amount of cleaved caspase 3 induced by neomycin was significantly reduced by pretreatment with citicoline. **(D)** Quantification of the western blot in **(C)**. **(E)** The mRNA levels of five apoptosis-related genes were analyzed by qRT-PCR normalized to *GAPDH* and presented as the fold of control levels. Data are shown as mean ± SD. *p < 0.05, **p < 0.01, ***p < 0.001. Scale bars = 20 μm.

We further studied the impact of citicoline on the expression of pro-apoptotic and anti-apoptotic factors in HEI-OC-1 cells after neomycin injury. Immunofluorescence staining indicated that the percentage of cleaved caspase 3-positive cells was significantly higher in the neomycin-only group compared with the undamaged controls (Figures 4A,B), but citicoline-treated cells showed a significantly reduced percentage of cleaved caspase 3-positive cells compared to the neomycin-only group (Figures 4A,B). Consistent with these results, we found that the protein levels of cleaved caspase 3 in HEI-OC-1 cells was significantly increased after neomycin treatment compared with the undamaged controls, and the protein levels were significantly reduced by citicoline pretreatment (Figures 4C,D). Furthermore, qRT-PCR analysis showed that the anti-apoptotic factor *Bcl-2* was significantly decreased in the neomycin-only group, while the expression of intrinsic and extrinsic pro-apoptotic marker genes, including *Bax*, *Casp3*, *Casp8*, and *Casp9*, was significantly higher compared to the undamaged controls (Figure 4E). In the citicoline-treated group, the expression levels of these intrinsic and extrinsic pro-apoptotic factors were significantly decreased, and expression of the anti-apoptotic factor *Bcl*-2 was significantly higher compared to the neomycin-only group (Figure 4E). Together, our results suggested that citicoline is involved in neomycin-induced HEI-OC-1 apoptosis by inhibiting the expression of both intrinsic and extrinsic apoptotic factors.

### Citicoline Attenuates Oxidative Stress in Cochlear HCs After Neomycin Injury

**FIGURE 5:**
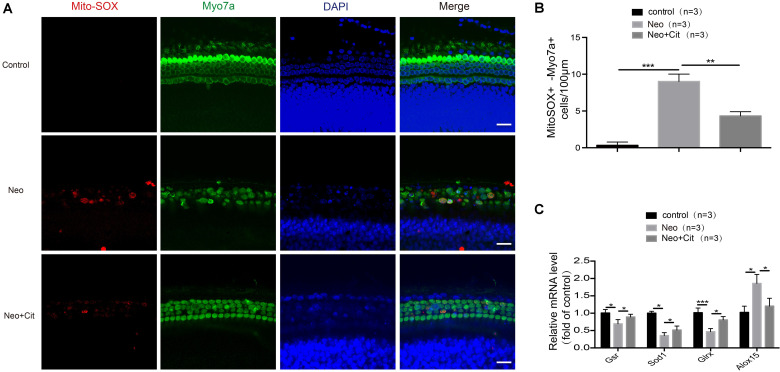
Citicoline attenuates oxidative stress in cochlear HCs after neomycin exposure. **(A)** Immunofluorescence staining with Mito-SOX Red and anti-Myo7A antibodies in the middle turn of the cochlea after different treatments. **(B)** Quantification of the numbers of Mito-SOX Red and Myo7A double-positive cells in **(A)**. **(C)** The mRNA levels of genes related to redox reactions were analyzed by qRT-PCR after neomycin and citicoline treatment and normalized to *GAPDH* and presented as the fold change compared to control levels. Data are shown as mean ± SD. *p < 0.05, **p < 0.01, ***p < 0.001. Scale bars = 20 μm.

In this experiment, we sought to determine the relationship between citicoline and oxidative stress in cochlear HCs. We dissected and cultured the basilar membranes from P3 mice and treated them with neomycin together with citicoline, and Mito-SOX Red was used to measure mitochondrial ROS levels in the cochleae. Quantification of Mito-SOX Red colocalization with Myo7A showed that the ROS levels were increased in the cochlea after neomycin treatment compared with the undamaged group, and the citicoline-treated cochleae showed significantly lower ROS levels compared to the neomycin-only group (Figures 5A,B).

We next performed qRT-PCR to measure the mRNA expression of redox-related genes in cochlear HCs after neomycin treatment. We found that the expression of the antioxidant genes *Gsr*, *Sod1*, and *Glrx* was significantly decreased in cochlear HCs after neomycin exposure compared with undamaged controls, and the pro-oxidant factor *Alox15* was significantly increased in the neomycin-only group (Figure 5C). We then measured the expression of these genes after treatment with citicoline. We found that the expression of the antioxidant genes *Gsr*, *Sod1*, and *Glrx* was significantly increased and the pro-oxidant factor *Alox15* was significantly reduced compared with the neomycin-only group (Figure 5C).

### Citicoline Increases the Mitochondrial Membrane Potential of HEI-OC-1 Cells After Neomycin Exposure

**FIGURE 6:**
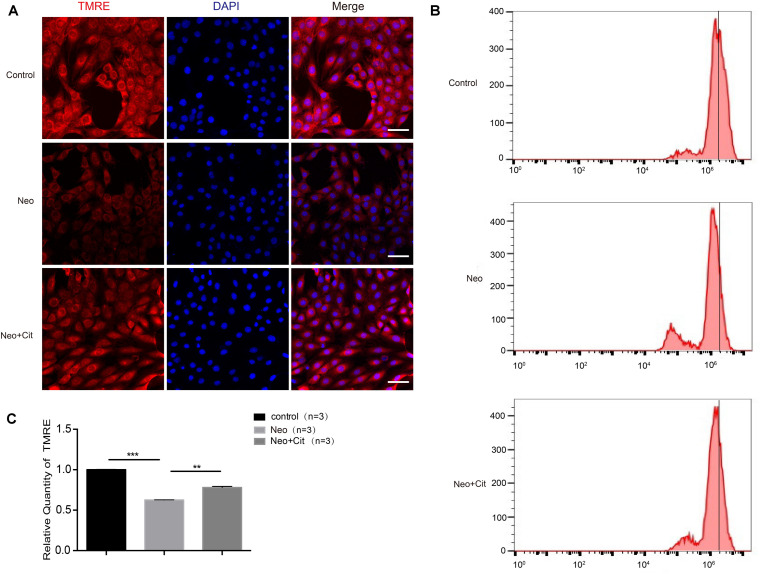
Citicoline increases the MMP of HEI-OC-1 cells after neomycin exposure. **(A)** The three groups of HEI-OC-1 cells were labeled using the TMRE staining kit. **(B)** Flow cytometry data confirmed the results in **(A)**. **(C)** Quantification of the flow cytometry results in **(B)**. The TMRE intensity was significantly decreased after neomycin exposure compared to the undamaged controls. In addition, the TMRE intensity was significantly increased in the citicoline treatment group compared to the neomycin-only group. Data are shown as mean ± SD. *p < 0.05, **p < 0.01, ***p < 0.001. Scale bars = 20 μm.

To further explore the mechanism behind the role of citicoline in neomycin-induced apoptosis of HEI-OC-1 cells, we used TMRE kits to measure changes in the MMP in HEI-OC-1 cells using immunofluorescence staining and flow cytometry analysis. The TMRE intensity was significantly decreased after neomycin treatment compared to the undamaged control group (Figures 6A–C), and the citicoline-treated cells showed a significantly greater TMRE intensity than the neomycin-only group (Figures 6A–C). These results demonstrated that citicoline protects HEI-OC-1 cells from apoptosis by inhibiting MMP dysfunction.

### Citicoline Inhibits Neomycin-Induced Oxidative Stress in HEI-OC-1 Cells

**FIGURE 7:**
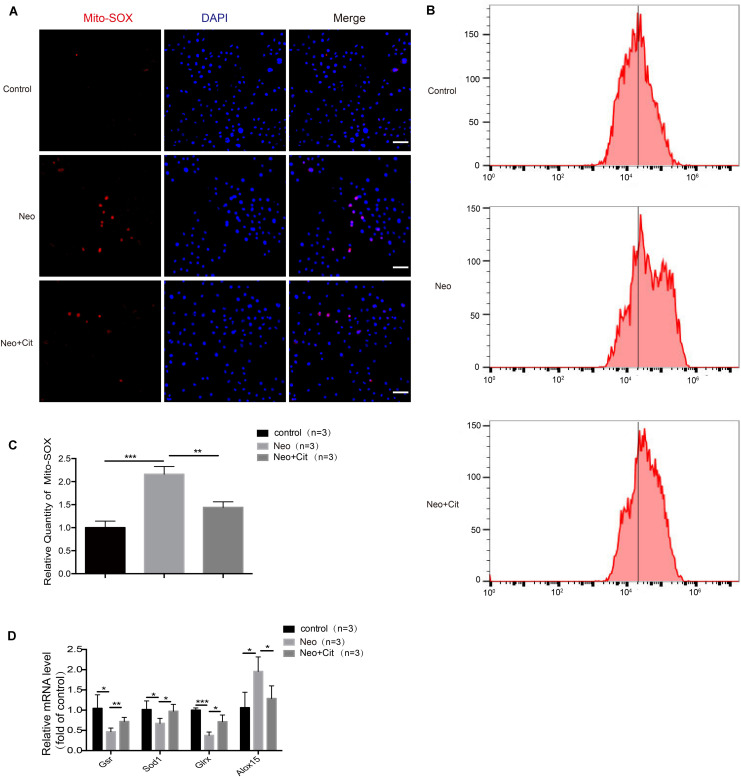
Citicoline modulates neomycin-induced oxidative stress in HEI-OC-1 cells. **(A)** The three groups of HEI-OC-1 cells were labeled using the Mito-SOX Red staining kit. **(B)** Flow cytometry data confirmed the results in **(A)**. **(C)** Quantification of the flow cytometry results in **(B)**. The ROS levels were significantly increased after neomycin treatment compared to the undamaged group. The ROS levels induced by neomycin were significantly reduced by pretreatment with citicoline. **(D)** The mRNA levels of genes related to redox reactions were analyzed by qRT-PCR and normalized to *GAPDH* and presented as the fold change compared to control levels. Data are shown as mean ± SD. *p < 0.05, **p < 0.01, ***p < 0.001. Scale bars = 20 μm.

We used Mito-SOX Red to evaluate mitochondrial ROS levels in HEI-OC-1 cells after neomycin treatment. Immunofluorescence and flow cytometry results showed that the ROS levels were significantly increased after neomycin treatment compared with the undamaged group and were significantly reduced in the citicoline-treated group compared with the neomycin-only group (Figures 7A–C).

To further verify our findings, we analyzed the mRNA expression of four redox-related genes by qPCR. The expression of the pro-oxidant factor *Alox15* increased after neomycin treatment compared with the undamaged controls, while the antioxidant genes *Gsr*, *Sod1*, and *Glrx* were significantly decreased in the neomycin-only group (Figure 7D). In addition, treatment with citicoline significantly upregulated the expression of the antioxidant genes *Gsr*, *Sod1*, and *Glrx* and reduced the expression of the pro-oxidant factor *Alox15* compared with the neomycin-only group (Figure 7D). Our results indicated that citicoline increased the expression of antioxidant genes and decreased the expression of pro-oxidant genes and thus reduced mitochondrial ROS levels in the cells and prevented apoptosis after neomycin injury.

### Citicoline Downregulates the Expression of VDAC1 in HEI-OC-1 Cells After Neomycin Exposure

**FIGURE 8:**
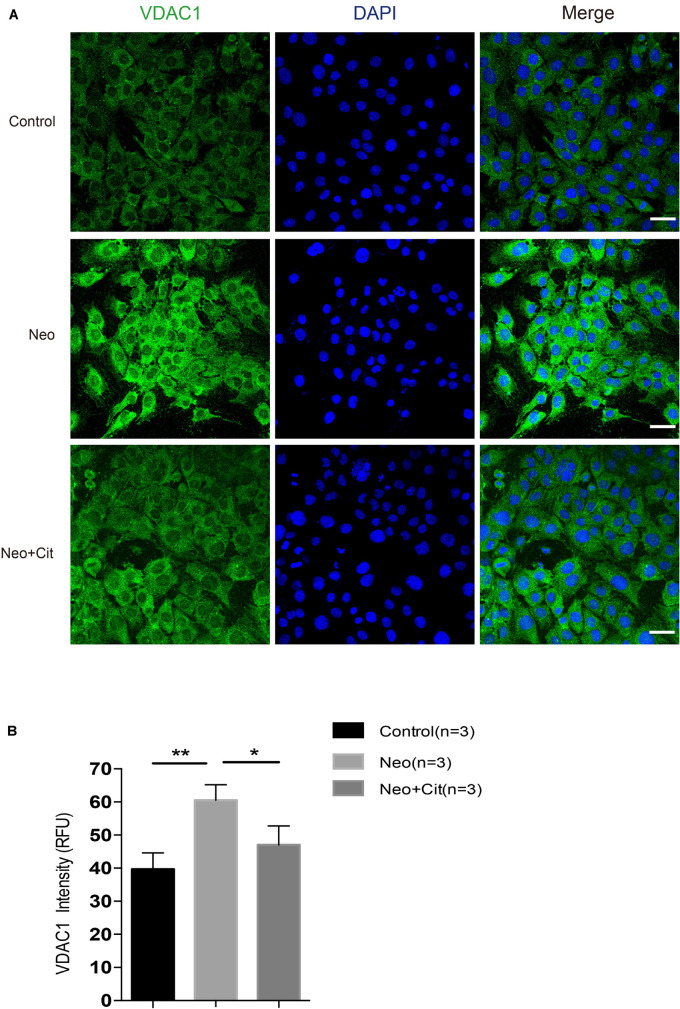
Citicoline downregulates the expression of the VDCA1 of HEI-OC-1 cells after neomycin exposure. **(A)** The three groups of HEI-OC-1 cells were labeled using the mitochondrial marker VDAC1 and DAPI double staining. **(B)** Quantitative analysis histogram of VDAC-1 staining intensity scanning in **(A)**. Data are shown as mean ± SD. *p < 0.05, **p < 0.01, ***p < 0.001. Scale bars = 20 μm.

VDAC1 is a major channel protein located in the outer mitochondrial membrane, and it plays an important regulatory role in the communication between the mitochondria and other parts of the cell. Compared with the undamaged group, the neomycin-only group was characterized by the obviously enhanced VDAC1-positive staining; however, treatment with citicoline significantly downregulated the mitochondrial fluorescence intensity (Figures 8A,B). The VDAC1 staining further confirmed that citicoline inhibits neomycin-induced oxidative stress by reducing mitochondrial ROS levels.

## Discussion

Ototoxic drugs are a common cause of sensorineural hearing loss, and aminoglycosides are the most commonly used ototoxic drugs. Cochlear HCs are susceptible to aminoglycoside-mediated cytotoxicity and cannot be regenerated once damaged, and thus aminoglycoside ototoxicity is usually associated with permanent sensorineural deafness (Nadol, 1993; Lazarou et al., 2015; Correia-Melo et al., 2017). Recent studies have reported that HCs in the mouse cochlea have a very limited ability to regenerate during the neonatal period, but this limited spontaneous HC regeneration is not sufficient to restore hearing ability once HCs are destroyed by aminoglycosides, and all regenerative ability is lost a few days after birth (Li et al., 2016; Ni et al., 2016; Waqas et al., 2016; Lu et al., 2017). To explore the mechanisms through which ototoxic drugs cause auditory sensory cell injury and to take appropriate control measures, it has become especially urgent to prevent and treat such diseases. Previous studies have shown that the toxic effects of aminoglycosides on auditory sensory cells are clearly associated with oxidative stress-induced cellular damage and the induction of apoptosis.

Citicoline, a nucleoside derivative, is an indispensable endogenous intermediate in the biosynthesis of phosphatidylcholine, which has been widely demonstrated to play a therapeutic role in the development of central nervous system injury and neurodegenerative diseases, and citicoline has specific effects on promoting brain function recovery and in promoting wakefulness (Wignall and Brown, 2014). Citicoline is a safe and well-tolerated drug without significant systemic side effects, and it has been shown that 100 μM of citicoline is not harmful to retinal cells (Davinelli et al., 2017), and even up to 1000 μM citicoline is not harmful to retinal neuroglial cells *in vitro* (Matteucci et al., 2014). We found that the protective effect of citicoline is dose dependent, and the viability of HEI-OC-1 cells was highest when the concentration of citicoline was 10 μM. We still observed the protective effect at a concentration of 100 μM, but the protective effect began to decline significantly when the concentration of citicoline reached 1 mM. Taken together, these results confirm that citicoline is safe for HEI-OC1 cells *in vitro*, which is consistent with the good tolerability profile for citicoline in clinical studies (Supplementary Figure S1).

Citicoline is an old drug, and we often ignore its other functions, such as whether it also protects HCs. Previous studies focusing on nerve cells found that citicoline plays a protective role through anti-oxidation and anti-apoptosis activities (Barrachina et al., 2002; Park et al., 2006), and we hypothesized that citicoline might also have an important protective effect against aminoglycoside-induced HC injury. In this study, we investigated the role of citicoline in both neomycin-induced injury in HEI-OC-1 cells and in cochlear HCs, and we found that citicoline significantly decreased apoptosis after neomycin injury in both contexts (Figures 1, 3).

Apoptosis occurs via both intrinsic and extrinsic pathways (Rybak and Kelly, 2003). The aminoglycosides are believed to induce apoptosis by releasing the apoptotic enzyme activation factor that subsequently activates caspases (Karasawa and Steyger, 2011). In this study, we found that cell death and apoptosis dramatically increased in both HEI-OC-1 cells and in cochlear HCs after neomycin injury, while citicoline significantly reduced the neomycin-induced cell death and apoptosis (Figures 2, 4). Furthermore, qRT-PCR analyses indicated that the expression of intrinsic and extrinsic pro-apoptotic genes (*Bax*, *Casp3*, *Casp8*, and *Casp9*) was significantly decreased in the citicoline-treated group after neomycin exposure, while the expression of the anti-apoptotic gene *Bcl-2* was significantly increased (Figures 2, 4). These results suggest that citicoline plays a critical anti-apoptotic role.

Bcl-2 family proteins regulate the integrity of the mitochondrial outer membrane and play an important role in determining mitochondria-mediated apoptosis. Pro-apoptotic proteins such as Bax translocate to the mitochondrial outer membrane to form oligomeric complexes when they encounter apoptotic signals, resulting in increased mitochondrial outer membrane permeabilization, Cytochrome-C release, and Caspase activation, while anti-apoptotic proteins such as Bcl-2 prevent this process (Martinou and Youle, 2011). Citicoline inhibits apoptosis in nerve cells by promoting the expression of anti-apoptotic factor *Bcl-2* and reducing the expression of the pro-apoptotic factor *Bax* (Levites et al., 2002). In addition, citicoline was also reported to protect the retina by increasing Bcl-2 expression (Schuettauf et al., 2006). Our results show that the expression of the anti-apoptotic gene *Bcl-2* was significantly increased and the expression of the pro-apoptotic gene *Bax* was significantly decreased after citicoline treatment. This suggested that citicoline can inhibit neomycin-induced HC injury by affecting the expression of *Bax* and *Bcl-2*.

Ototoxic drugs cause hearing loss by inducing HC apoptosis, primarily by altering the MMP of the mitochondria (Huang et al., 2000; Wu et al., 2002; Sun et al., 2015; Guan et al., 2016; He et al., 2016; Yu et al., 2017). Mitochondria play an important role in cell metabolism, and aminoglycoside-induced apoptosis is closely related to mitochondrial dysfunction, which leads to decreased MMP and increased ROS (Joza et al., 2001; Chipuk et al., 2004; Coffin et al., 2013; Sun et al., 2014, 2015; Mei et al., 2015). The accumulation of ROS in the mitochondria is an important trigger of apoptosis, and it has been reported that ROS play an important role in noise-induced and ototoxic drug-induced HC damage and hearing loss (Sun et al., 2014, 2015; Chen et al., 2015). Previous studies have shown that ROS accumulation triggers mitochondrial depolarization, changes mitochondrial membrane permeability, and induces apoptosis (Rybak et al., 2007; Nemec and Khaled, 2008; Chen et al., 2015; Sun et al., 2015). In the present study, we demonstrated that citicoline significantly increased the MMP of HEI-OC-1 cells and decreased ROS levels in HEI-OC-1 cells and in cochlear HCs after neomycin exposure (Figures 5–7), suggesting that citicoline alleviates mitochondrial dysfunction in both cell types after neomycin exposure.

In response to aminoglycoside-induced ROS accumulation, antioxidant genes are upregulated to counteract this accumulation. Therefore, the balance between oxidant and antioxidant gene expression is critical for the rate of ROS accumulation, and many genes coordinate with each other to regulate the balance between the production and scavenging of ROS. Administration of ROS-scavenging antioxidants (Sha and Schacht, 2000; Kawamoto et al., 2004; Kim et al., 2009; Ding et al., 2013), as well as inhibition of oxidase (Kim et al., 2010), can reduce the ROS production, thus attenuating the subsequent HC death in ototoxic drug-treated cochleae. In this study, the expression of antioxidant genes was significantly decreased and the expression of pro-oxidation factors was significantly increased after neomycin exposure compared with undamaged controls. We also found that citicoline significantly increased the expression of several key antioxidant genes such as *Gsr*, *Sod1*, and *Glrx* and decreased the expression of the pro-oxidation factor *Alox15* (Figures 5, 7). Overall, these results indicate that citicoline increases the expression of antioxidant genes, thus leading to decreased ROS levels and preventing neomycin-mediated mitochondrial dysfunction and apoptosis in HEI-OC-1 cells and cochlear HCs.

In our study, the expression of antioxidant genes was significantly downregulated after neomycin exposure. We consider that increased ROS activates the cell defense mechanism in the early stage of cell damage, thus activating the cell antioxidant mechanism and up-regulating antioxidant factors to clear the ROS. However, with the aggravation of cell damage, aminoglycoside exposure induces large increases of ROS in the cochlear HCs that overwhelm the cellular defense mechanisms (Joza et al., 2001; Esterberg et al., 2016). This leads to the imbalance between intracellular oxidation and antioxidation resulting in a significant down-regulation of antioxidant factors and a significant increase in oxidant factors. Because we also previously found that the expression of antioxidant genes decreased after neomycin damaged hair cells (Chen et al., 2015; He et al., 2017), we hypothesize that the downregulation of antioxidant factors is related to the special sensitivity of HCs to aminoglycoside antibiotics, but this still needs further study.

VDAC is a major channel protein located in the outer mitochondrial membrane. It is well recognized that VDAC is involved in many physiological and pathophysiological processes, including Ca2+ homeostasis (Shoshan-Barmatz et al., 2006, 2010), energy metabolism (Shoshan-Barmatz et al., 2006; Saks et al., 2010), and cell apoptosis (Tsujimoto and Shimizu, 2002; Shoshan-Barmatz et al., 2010). VDAC has three isoforms (VDAC1-3), and VDAC1 is the main isoform mediating cell functions (De Stefani et al., 2012) and plays an important role in regulating intracellular ROS generation and subsequent apoptotic events (Chen et al., 2014). It is necessary for ROS to cross the outer mitochondrial membrane when released from the mitochondria to the cytoplasm, and this process is mediated by VDAC1. Increased intracellular ROS generation can be mostly suppressed by VDAC1 inhibitors, and thus VDAC1 appears to play a dominant role in regulating ROS generation (Chen et al., 2014). In our study, we demonstrated that citicoline significantly downregulated VDAC1 in HEI-OC-1 cells after neomycin exposure (Figure 8), thus leading to decreased ROS levels and inhibition of apoptosis.

Sirtuin 1 (SIRT1), the most conserved member of the NADC-dependent protein deacetylase family, has been shown to have protective effects in various common neurodegenerative disorders (Herskovits and Guarente, 2014). Citicoline has been recently shown to increase SIRT1 protein expression, and this is strongly related to its neuroprotective activities (Hurtado et al., 2013). A previous study showed that increased SIRT1 protects against cisplatin-induced damage to HCs (Xiong et al., 2019). Therefore, we will further study whether the protective effect of citicoline on neomycin-induced HC damage works through SIRT1 activation.

In summary, we first used citicoline on hair cells and clarified the importance of the drug on ear hair cells for the first time. Our study provides the first report that citicoline protects auditory HCs against neomycin injury by preventing mitochondrial dysfunction and the upregulation of antioxidant genes, thus leading to decreased ROS levels and preventing apoptosis. This study therefore provides experimental evidence for the potential clinical application of citicoline to prevent aminoglycoside-induced auditory HC damage.

## Data Availability Statement

The original contributions presented in the study are included in the article/Supplementary Material, further inquiries can be directed to the corresponding author/s.

## Ethics Statement

The animal study was reviewed and approved by the Animal Care and Use Committee of Southeast University. Written informed consent was obtained from the owners for the participation of their animals in this study.

## Author Contributions

ZZ, XF, HL, and JC contributed equally to this work. All the authors made substantial and direct intellectual contributions to the work and approved it for publication.

## Conflict of Interest

The authors declare that the research was conducted in the absence of any commercial or financial relationships that could be construed as a potential conflict of interest.

## Supplementary Material

FIGURE S1 Citicoline promotes HEI-OC1 cells survival after neomycin exposure. **(A)** The CCK-8 kit measured the cell viability after treatment with different neomycin concentrations (1–20 mM) for 24 h. **(B)** The cell viability was measured by the CCK-8 kit after treatment with 10 mM neomycin for different times (0, 6, 12, 24, 48 h). **(C)** The cell viability after neomycin exposure was measured with the CCK-8 kit after pretreatment with different citicoline concentrations (1, 10, 100 μM, 1 mM, 2 mM) for different times (6, 12, 24 h). **(D)** CCK-8 result of HEI-OC1 cells pre-treated with different citicoline concentrations for 6 h after neomycin exposure. **(E)** CCK-8 result of HEI-OC1 cells pre-treated with different citicoline concentrations for 12 h after neomycin exposure. **(F)** CCK-8 result of HEI-OC1 cells pre-treated with different citicoline concentrations for 24 h after neomycin exposure. Data are shown as mean ± SD. **p* < 0.05, ***p* < 0.01, ****p* < 0.001.
