# Supplementary figures and images for: Citicoline Protects Auditory Hair Cells Against Neomycin-Induced Damage
Source: Front Cell Dev Biol. 2020 Aug 31;8:712. doi: 10.3389/fcell.2020.00712 (PMC7487320; doi:10.3389/fcell.2020.00712)

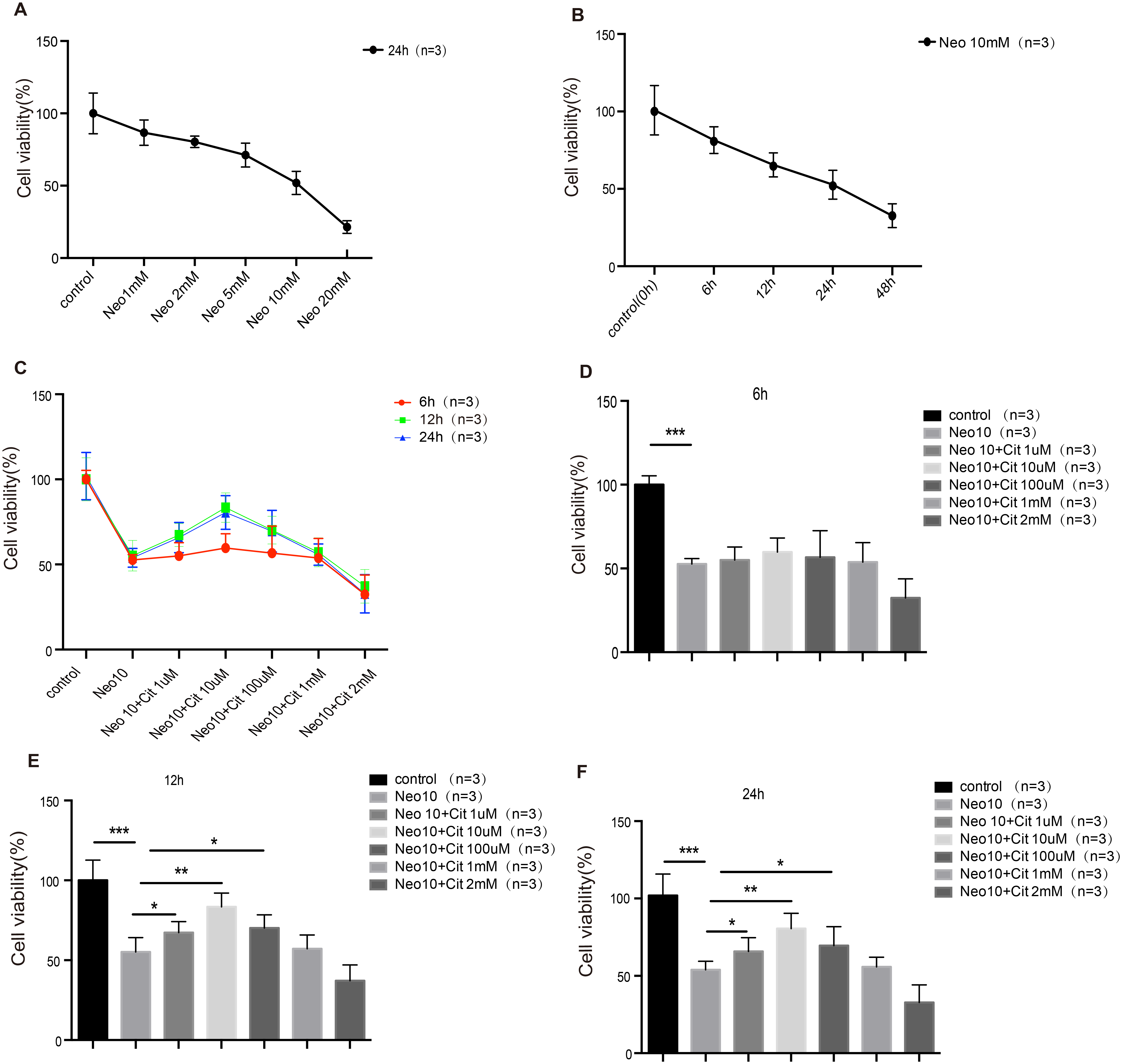

Supplement: FIGURE S1 — Citicoline promotes HEI-OC1 cells survival after neomycin exposure. (A) The CCK-8 kit measured the cell viability after treatment with different neomycin concentrations (1–20 mM) for 24 h. (B) The cell viability was measured by the CCK-8 kit after treatment with 10 mM neomycin for different times (0, 6, 12, 24, 48 h). (C) The cell viability after neomycin exposure was measured with the CCK-8 kit after pretreatment with different citicoline concentrations (1, 10, 100 μM, 1 mM, 2 mM) for different times (6, 12, 24 h). (D) CCK-8 result of HEI-OC1 cells pre-treated with different citicoline concentrations for 6 h after neomycin exposure. (E) CCK-8 result of HEI-OC1 cells pre-treated with different citicoline concentrations for 12 h after neomycin exposure. (F) CCK-8 result of HEI-OC1 cells pre-treated with different citicoline concentrations for 24 h after neomycin exposure. Data are shown as mean ± SD. *p < 0.05, **p < 0.01, ***p < 0.001. [file Image_1.jpeg]
